# Expression and up-regulation of interleukin-6 in oesophageal carcinoma cells by n-sodium butyrate

**DOI:** 10.1038/sj.bjc.6690571

**Published:** 1999-07

**Authors:** L-S Wang, K-C Chow, C-W Wu

**Affiliations:** Divisions of Chest and General Surgery, Department of Surgery, and Department of Education and Research, Veterans General Hospital in Taipei and National Yang-Ming University, Taipei, Taiwan, ROC

**Keywords:** oesophageal carcinoma, interleukin-6, sodium butyrate, up-regulation

## Abstract

Recently, the serum level of interleukin (IL)-6 has been shown to correlate with disease progression and prognosis of cancer patients. However, the available information about the source and the pathophysiological regulation of IL-6 in cancer cells is limited. Thus, in this study, we tried to identify the source and the clinical roles of serum IL-6 in patients with oesophageal squamous cell carcinoma (ESCC), and then further to characterize the biological regulation of IL-6 in ESCC cell lines. Sera and tissue specimens from 80 consecutive patients with ESCC were collected between 1993 and 1997. Additionally, three ESCC cell lines were used for in vitro study. The concentration of serum IL-6 was measured by enzyme-linked immunosorbent assay (ELISA), and correlated the survival time with measured IL-6 level. Expressions of IL-6, IL-6Rα (IL-6 receptor alpha) and gp130 in pathological sections and cell lines were characterized by immunological staining. Detection of IL-6 mRNA was determined by in situ hybridization (ISH) and reverse transcription-polymerase chain reaction (RT-PCR). Up-regulation of IL-6 by n-sodium butyrate (n-BT) was studied in ESCC cell lines. The levels of serum IL-6 in patients with ESCC were significantly higher than those in the healthy controls. Serum levels of IL-6 were also shown to correlate with disease progression and survival. However, sCD8 levels and lymphocyte counts in the peripheral blood were not parallel to the changed pattern of serum IL-6. In pathological sections and ESCC cell lines, message of IL-6 was identified by ISH in cancer cells. Expression of IL-6 mRNA was further confirmed with RT-PCR in ESCC cell lines. Although IL-6 was detected in some ESCC cell lines, IL-6 gene expression and protein production could be induced or enhanced by n-BT treatment in all three cell lines. The serum levels of IL-6 are frequently elevated at diagnosis of ESCC, and are associated with poor prognosis. IL-6 that could be produced by cancer cells is up-regulated by n-BT. © 1999 Cancer Research Campaign


*British Journal of Cancer* (1999) **80**(10), 1617–1622
© 1999 Cancer Research Campaign
Article no. bjoc.1999.0571

### Expression and up-regulation of interleukin-6 in oesophageal carcinoma cells by n-sodium butyrate

**L-S Wang[1], K-C Chow[2] and C-W Wu[3]**

Divisions of [1]Chest and [2]General Surgery, Department of Surgery, and [3]Department of Education and Research, Veterans General Hospital in Taipei and National Yang-Ming University, Taipei, Taiwan, ROC

**Summary** Recently, the serum level of interleukin (IL)-6 has been shown to correlate with disease progression and prognosis of cancer patients. However, the available information about the source and the pathophysiological regulation of IL-6 in cancer cells is limited. Thus, in this study, we tried to identify the source and the clinical roles of serum IL-6 in patients with oesophageal squamous cell carcinoma (ESCC), and then further to characterize the biological regulation of IL-6 in ESCC cell lines. Sera and tissue specimens from 80 consecutive patients with ESCC were collected between 1993 and 1997. Additionally, three ESCC cell lines were used for in vitro study. The concentration of serum IL-6 was measured by enzyme-linked immunosorbent assay (ELISA), and correlated the survival time with measured IL-6 level. Expressions of IL-6, IL-6Rα (IL-6 receptor alpha) and gp130 in pathological sections and cell lines were characterized by immunological staining. Detection of IL-6 mRNA was determined by in situ hybridization (ISH) and reverse transcription-polymerase chain reaction (RT-PCR). Up-regulation of IL-6 by n-sodium butyrate (n-BT) was studied in ESCC cell lines. The levels of serum IL-6 in patients with ESCC were significantly higher than those in the healthy controls. Serum levels of IL-6 were also shown to correlate with disease progression and survival. However, sCD8 levels and lymphocyte counts in the peripheral blood were not parallel to the changed pattern of serum IL-6. In pathological sections and ESCC cell lines, message of IL-6 was identified by ISH in cancer cells. Expression of IL-6 mRNA was further confirmed with RT-PCR in ESCC cell lines. Although IL-6 was detected in some ESCC cell lines, IL-6 gene expression and protein production could be induced or enhanced by n-BT treatment in all three cell lines. The serum levels of IL-6 are frequently elevated at diagnosis of ESCC, and are associated with poor prognosis. IL-6 that could be produced by cancer cells is up-regulated by n-BT.

**Keywords:** oesophageal carcinoma; interleukin-6; sodium butyrate; up-regulation

Oesophageal carcinoma is a common cancer in China and Taiwan (Wu et al, 1980; Wang et al, 1992). Its annual incidence rate varies from $3/10^5$ to $25/10^5$, depending upon genetic vulnerability, diet and environmental factors. Surgery is a curative treatment for the early stage oesophageal squamous cell carcinoma (ESCC) (Katlic et al, 1990; Wang et al, 1996), however, most patients present with advanced disease. Furthermore, poor prognosis is compounded with the rapid growth and spread of cancer cells as well as dysphagia-associated malnutrition and cachexia (Katlic et al, 1990; Strassmann and Jacob et al, 1992). The effort of multiple therapeutic modalities was not beneficial for patients at late stage. Therefore, a method for identifying the disease progression of ESCC and the potential of cancer cell spreading is important to commence the early treatment and to improve survival.

Interleukin-6 (IL-6), besides being a multi-functional cytokine with a wide spectrum of immunological activities (Kishimoto, 1990), is a potent endogenous pyrogen (Gauldie et al, 1987; Castell et al, 1990) and a potential mediator in the development of cancer cachexia (Strassmann and Fong et al, 1992). It has been detected in primary squamous cell carcinomas, adenocarcinomas and sarcomas (Tabibzadeh et al, 1989), as well as tumour cell lines derived from melanoma (Lee et al, 1992), glioblastoma (VanMeir et al, 1990; Stephanou et al, 1992), lung cancer (Takizawa et al, 1993; Takeuchi et al, 1996; Inoue et al, 1997), ovarian cancer (Watson et al, 1990) and cervical cancer (Eustace et al, 1993). Clinically, serum levels of IL-6 were also demonstrated to correlate with adverse prognosis in patients with metastatic renal cell carcinoma (Blay et al, 1992), ovarian cancer (Scambia et al, 1995), gastric cancer (Kabir and Daar, 1995; Wu et al, 1996), lung cancer (Yanagawa et al, 1995; Wojciechowska-Lacka et al, 1996) and Hodgkin's lymphoma (Seymour et al, 1997).

In vitro, IL-6 was further shown to act as an autocrine growth factor in stimulating the proliferation of multiple myeloma (Kawano et al, 1988), renal cell carcinoma (Mini et al, 1989) and non-Hodgkin's lymphoma cells (Ni and O'Neill, 1992). Recently, Oka et al (1996) showed that ESCC could also express both IL-6 and its receptor (IL-6R). It is conceivable that IL-6 could have pathogenetic significance in disease progression of this malignancy. Nonetheless, the induction effect of IL-6 on tumour cell growth might not be invariably obtained (Yanagawa et al, 1995). The difference could be in part due to the marked heterogeneity of gene expression in a tumour cell population. In part, IL-6 gene may not be expressed constitutively. It might be subjected to a pathophysiological regulation in cancer cells. A recent study has indicated that both urinary butyrate concentration and IL-6 level were increased in AIDS patients with significant weight loss (Stein et al, 1997), a condition that is similar to what a cancer

*Received 1 October 1998*
*Revised 9 December 1998*
*Accepted 20 January 1999*

*Correspondence to:* L-S Wang, Division of Thoracic Surgery, Department of Surgery, Veterans General Hospital in Taipei, #201 Sec. 2, Shih-Pai Road, Taipei 11217, Taiwan, ROC

patient could develop during cachexia (Strassmann et al, 1992). Butyrate is a short-chain fatty acid produced by intestinal bacteria that could escape from the host immunosurveillance in intestine during disease progression. Serum butyrate concentration could therefore reflect the intestinal situation, and it is reasonable to ask whether butyrate could have any effect on IL-6 expression at the advanced stage of ESCC.

In this study, we examined serum levels of IL-6 in 80 ESCC patients and relationships among serum IL-6 level, clinicopathological factors and survival following surgery. Furthermore, we indentified the source of serum IL-6 by examinations of IL-6, IL-6 mRNA and IL-6 receptors in tumour specimens and the ESCC cell lines, and characterized the physiological regulation of IL-6 expression by n-BT. The role of IL-6 in autocrine growth regulation for ESCC would then be discussed.

### METHODS

### Human sera, tissue specimens and tumour cell lines

From February 1993 to November 1997, sera and tissue specimens from 80 consecutive patients with newly diagnosed oesophageal cancer were collected. All patients had pathologically confirmed oesophageal squamous cell carcinoma (ESCC). The preoperative work-up consisted of oesophagoscopy with biopsy, oesophagogram, chest radiography, sonogram of the abdomen, computerized tomography (CT) scan of the chest and radionuclide scanning of whole body bone. All patients underwent en bloc oesophagectomy with locoregional lymphadenectomy through a right thoracotomy and laparotomy with reconstruction using the stomach through a retrosternal route, and cervical oesophagogastrostomy (Liu et al, 1998; Wang et al, 1998). Concurrent chemoradiotherapy would be administered after surgery for patients with stages beyond IIb (Wang et al, 1996, 1998). However, none received neoadjuvant therapy in the present series. After treatment, all patients were followed as routine. Stage of disease progression was classified according to the Union International Centre Cancer system. All stage IV patients were due to distant lymph node metastasis (cervical, coeliac, or para-aortic regions, etc.). Sera and peripheral blood cells were collected from patients at the time of diagnosis, four times at 3-month intervals, and then 6-month intervals following treatment. Sera from 103 healthy donors with an equivalent distribution of age and sex were collected as normal control. The Medical Ethical Committee approved the protocol, and the study was strictly following their guidelines. Written informed consent was obtained from every patient. A single-blind procedure was followed to carry out enzyme-linked immunosorbent assay (ELISA), immunostaining (IMS) and in situ hybridization (ISH) protocols. Three ESCC cell lines (48T, 81T and 146T, obtained from Dr CP Hu, Department of Medical Research, Veterans General Hospital–Taipei) were used for in vitro study. Up-regulation of IL-6 by n-BT was studied in ESCC cell lines. The expression of IL-6 mRNA in the cancer cell lines was confirmed in ESCC cells with ISH and reverse transcription polymerase chain reaction (RT-PCR) method. Cells were grown at 37°C in a monolayer in RPMI-1640 supplemented with 10% fetal calf serum, 100 IU $ml^{-1}$ penicillin and 100 μg $ml^{-1}$ streptomycin.

### ELISA

The concentration of serum IL-6 was determined by ELISA with Quantikine HS human IL-6 (R & D Systems Inc., Minneapolis, MN, USA). Briefly, 50 μl of assay diluent were added to a well that was pre-coated with monoclonal antibody specific to IL-6, before addition of 200-μl sample. The reaction was incubated at room temperature for 16 h. The microtitre plate was washed with wash buffer four times, and 200 μl of alkaline phosphatase-conjugated polyclonal antibody specific to IL-6 was then added. The reaction was further incubated at room temperature for 6 h. After washing four times with wash buffer, 50 μl of NADPH was added, and reaction was incubated at room temperature for 60 min. A positive reaction was identified by developing with amplifier solution containing diaphorase and INT-violet at room temperature for 30 min, and by reading at $A_{490}$ nm (MRX, Dynatech Laboratories Inc., Chantilly, VA, USA). Each individual sample was analysed in duplicate. Samples with overscaled values were diluted before further determination. Additionally, levels of sCD8 was also measured by ELISA with CD8 test kit (T cell Diagnostics, Cambridge, MA, USA).

### Immunological staining

Immunological staining was used to detect the expressions of IL-6 and its receptors, IL-6Rα and gp 130 (IL-6 signal transducer), in the cancer cell lines and pathological sections. Antibodies used for immunological staining were specific to IL-6, IL-6Rα (R&D Systems Inc., Minneapolis, MN, USA) and gp130 (Santa Cruz Biotechnology Inc., Santa Cruz, CA, USA) respectively. Immunological staining was performed by an immunoperoxidase method as previously described (Chiu et al, 1997).

### ISH

A non-isotopic method, using a mixture of fluorescein isothiocyanate (FITC)-labelled IL-6 antisense oligonucleotides, was used to determine the expression of IL-6 mRNA in cell lines and pathological sections (Chow et al, 1992). The probe sequences were (Wang et al, 1988): 5′-GGACAGGTTTCTGACCAGA-AGAA GGAATGCCC-3′ (IL-6 mRNA, 750–719) 5′-ACT-GCAGGAACTCCTTAAAGCTGCGCAGAA-3′ (IL-6 mRNA, 660–631) 5′-TCACCAGGCAAGTCTCCTCATTGAATCCAG-3′ (IL-6 mRNA, 390–361). Hybridization products were visualized by using alkaline phosphatase-conjugated polyclonal antibodies to FITC (Amersham International, Buckinghamshire, UK) and chromogen NBT/BCIP (Sigma, St Louis, MO, USA). Positive staining was identified microscopically as brownish blue granules at the site of hybridization.

### RNA extraction and signal amplification (RT-PCR)

Total RNA was extracted from $1 \times 10^7$ ESCC cells using SNAP RNA column (Invitrogen Corporation, San Diego, CA, USA). Following spectrophotometric determination of total RNA yield, cDNA was synthesized by oligo dT primer and AMV reverse transcriptase. An aliquot of cDNA was then subjected to 35 cycles of PCR using standard procedure denaturing at 94°C for 1 min, hybridizing at 52°C for 30 s, and elongating at 72°C for 1.2 min. The primers were 5′-CTGGATTCAATGAGGAGACTTGC-3′ (IL-6 mRNA, 361–383, sense primer) and 5′-GGACAGGTTTCT-GACCAGAAG-3′ (IL-6 mRNA, 750–730, antisense primer). The amplified products were resolved in a 2.5% agarose-ethidium bromide gel. Specificity of the 390 base-pair amplified product was confirmed by DNA sequencing (ABI Prism, Foster City, CA, USA).

 *© 1999 Cancer Research Campaign*

### Statistical analysis

The relation between serum level of IL-6 and clinicopathologic parameters (age, stage, lymph node metastasis, tumour depth) was analysed by $\chi^2$ analysis (or Fisher's exact test when expected number of any cell was smaller than or equal to five cases). Survival curves were plotted with the method of Kaplan–Meier (Kaplan and Meier, 1958). A total of 59 patients entered the survival curve analysis because of the necessity of an adequate follow-up period (⩾12 months). Statistical differences in survival between different groups was compared by the log-rank test (Peto and Pike, 1973). Statistical analysis was performed using SPSS statistical software (Chicago, IL, USA).

### RESULTS

### Elevated serum concentrations of IL-6 in patients with ESCC

The average age of male patients (*n* = 76) was 63.39 ± 10.9 years and that of female patients (*n* = 4) was 62.25 ± 3.86 years. Preoperative serum IL-6 levels in 93.8% (75/80) of ESCC patients (12.8 ± 12.1 pg $ml^{-1}$) were above normal average (2.8 ± 0.9 pg $ml^{-1}$, *n* = 103). Among these patients, 61.3% (46/75) were above 9 pg $ml^{-1}$. ESCC patients were then divided into two groups based on the preoperative serum levels of IL-6. In group A (*n* = 46), IL-6 levels were ≥ 9 pg $ml^{-1}$, and in group B (*n* = 34), IL-6 levels were < 9 pg $ml^{-1}$. No significant age difference was found between group A (64.06 ± 10.0 years) and group B (62.35 ± 11.35 years). By $\chi^2$ analysis, among the TNM categories only lymph node metastasis (N status) correlated with serum IL-6 level and that accounted for the overall correlation of the TNM classification with serum IL-6 (Table 1). In 15 patients with persistent high levels of serum IL-6 or a marked increase of serum IL-6 level within a short interval (0.5–3 months) following oesophagectomy, all patients developed tumour of recurrence within 12 months after surgery. On the other hand, most patients with low preoperative serum levels of IL-6 (*n* = 21) or those with a decrease of serum IL-6 level following oesophagectomy (*n* = 23) could remain disease-free for 12–23 months (30/44, 68.2%). Relationship between serum IL-6 level and survival was shown in Figure 1. The cumulative 2-year survival rate for patients in group A was 26.3% and that in group B was 66.7%. The group B patients had a superior survival rate compared to the group A patients (*P* = 0.0086).

**Table 1** Relationship between serum levels of IL-6 and clinicopathological factors

| | Group[a] | | | |
|---|---|---|---|---|
| Clinicopathological factors | A | B | $\chi^2$ | *P* |
| Lymph node metastasis | | | | |
| Negative | 14 | 21 | 7.733 | 0.0056 |
| Positive | 32 | 13 | | |
| Tumour depth | | | | |
| No invasion to adventitia | 11 | 16 | 5.004 | 0.086 |
| Invasion to adventitia | 28 | 13 | | |
| Invasion to adjacent organ | 7 | 5 | | |
| Distant metastasis | | | | |
| Negative | 33 | 30 | 3.312 | 0.081 |
| Positive | 13 | 4 | | |
| Stage (TNM) | | | | |
| I | 3 | 10 | 12.85 | 0.005 |
| II | 9 | 11 | | |
| III | 15 | 8 | | |
| IV | 19 | 5 | | |

[a]Groups were divided by serum levels of IL-6. In group A, IL-6 levels were ≥ 9 pg $ml^{-1}$, and in group B, IL-6 levels were < 9 pg $ml^{-1}$.

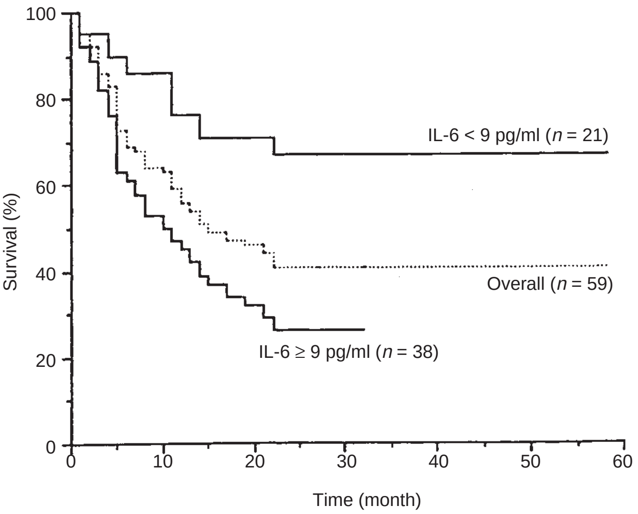

**Figure 1** Comparison of Kaplan–Meier product limit estimates of overall survival and survival analysis between group A (serum IL-6 levels were ≥ 9 pg $ml^{-1}$) and group B (serum IL-6 levels were <9 pg $ml^{-1}$). Survival difference between groups was compared by the log rank test. *P* = 0.0086

### Expression of IL-6 and IL-6Rα in pathological sections and cancer cell lines

Pathological sections and cultured cells were used for ISH to determine the expression of IL-6 mRNA. In Figure 2A, the hybridized products were identified in tumour cells. IL-6 mRNA was strongly expressed in 37.5% (30/80) of the pathological sections. IL-6Rα and gp130, however, were detected in 88.7% (71/80) of pathological sections. In the neighbouring lymphocytes of the pathological sections, on the other hand, IL-6 mRNA was only detected in three cases (3.8%). Levels of sCD8 and lymphocyte counts in the peripheral blood were not parallel to the change pattern of IL-6 either (data not shown). All patients with strong expression of IL-6 (*n* = 30) in pathological sections had a high level of serum IL-6 (≥ 9 pg $ml^{-1}$). These data indicate that tumour cells could be one of the major source of elevated serum IL-6 in patients with ESCC. The striking differences in disease progression, lymph node involvement and patients' survival rate suggested that IL-6 expression may vary among different patients, although the physiological factors remain to be determined.

Interestingly, there was a substantial increase of IL-6 expression when ESCC cell lines were treated with n-BT (Figure 2B,C). This issue was clarified when IL-6 mRNA was examined in n-BT-treated and control cells by RT-PCR (Figure 3). The difference was further extrapolated by a serial dilution of cDNA before PCR. The consequent determination of IL-6 content in the conditioned media of ESCC cell lines supported such observations. A marked increase of IL-6 in supernatant was detected in 81T cells (protein from 15.5 pg $ml^{-1}$ to 244 pg $ml^{-1}$, 64-fold increase in

© 1999 Cancer Research Campaign 

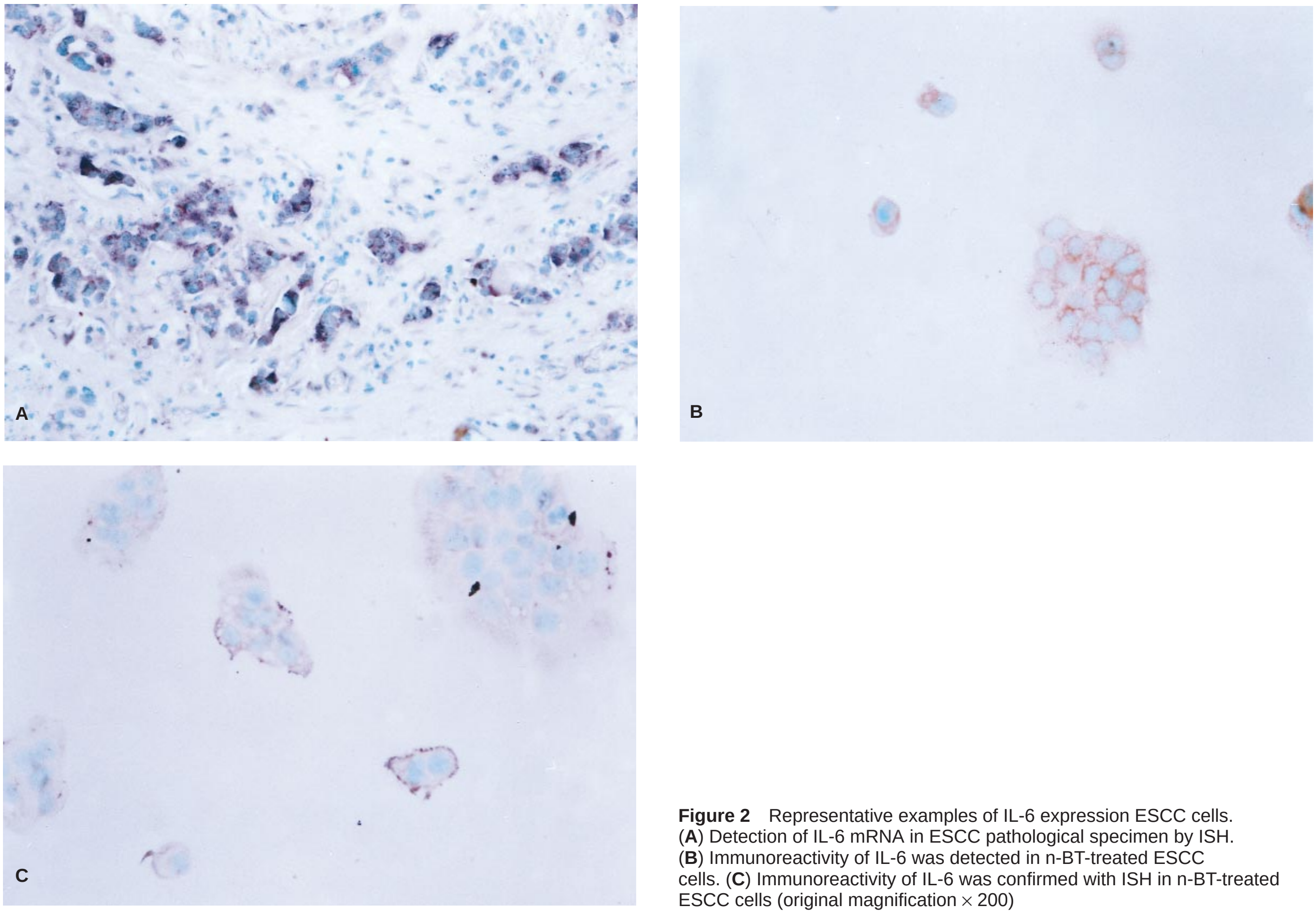

**Figure 2** Representative examples of IL-6 expression ESCC cells. (**A**) Detection of IL-6 mRNA in ESCC pathological specimen by ISH. (**B**) Immunoreactivity of IL-6 was detected in n-BT-treated ESCC cells. (**C**) Immunoreactivity of IL-6 was confirmed with ISH in n-BT-treated ESCC cells (original magnification × 200)

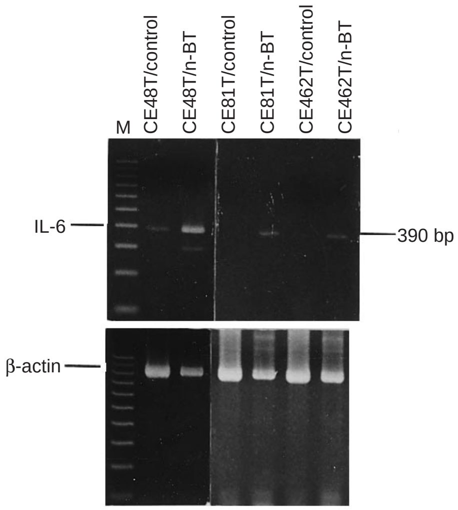

**Figure 3** Up-regulation of IL-6 mRNA in human oesophageal carcinoma cell lines by n-BT. IL-6 mRNA was detected by RT-PCR. A 390-bp human IL-6-specific sequence (upper panel) and a 900-bp β-actin sequence (lower panel) were amplified from total RNA isolated from human ESCC cell lines, analysed by agarose-ethidium bromide gel electrophoresis cDNA). Smaller changes of IL-6 were observed in 48T (protein from 6.9 pg $ml^{-1}$ to 13.4 pg $ml^{-1}$, fourfold increase in cDNA) and 146T cells (protein from undetectable to 2.6 pg $ml^{-1}$, 16-fold increase in cDNA). These results showed that n-BT could play a role in the upregulation of IL-6 expression in ESCC cells.

### DISCUSSION

Coexpression of IL-6 and IL-6 receptor (IL-6R) in cancer cells has, in recent years, provoked a hypothesis of autocrine growth factor mechanism (Kawano et al, 1988; Miki et al, 1989; Ni and O'Neill, 1992). IL-6 mediates its effects through two membrane proteins, a ligand-binding protein molecule (IL-6R) (Yamasaki et al, 1988) and a non-ligand-binding signal transducer (gp130) (Taga et al, 1989). Current evidence suggests that independent of direct interaction with T lymphocytes and natural killer cells IL-6 could promote tumour cell proliferation in several tumour cell lines (Kawano et al, 1988; Miki et al, 1989; VanMeir et al, 1990; Watson et al, 1990; Ni and O'Neill, 1992; Eustace et al, 1993; Oka et al, 1996; Takeuchi et al, 1996). Murine model as well as a study of human cervical carcinoma xenograft further support that IL-6 could promote tumour progression (Tamura et al, 1995). Besides having the ability to impair natural killer functions (Tanner and Tosato, 1991), IL-6 was shown to be a potent endogenous pyrogen (Gauldie et al, 1987; Castell et al, 1990) and a potential mediator in the development of cancer cachexia (Strassmann et al 1992*b*; Castell et al, 1990; Strassman et al, 1992*a*). In spite of these observations, the mechanism connecting autocrine tumour growth and development of cancer cachexia remains to be elucidated.

An elegant study by Oka et al (1996) has demonstrated that oesophageal cancer could express both IL-6 mRNA and IL-6Rα.

© 1999 Cancer Research Campaign

High concentrations of IL-6 in the tumour homogenates further suggest that tumour cells may produce IL-6. It should be clear that the tumour cells produce IL-6 if they are seen by ISH. Not only have we confirmed their finding by showing expression of IL-6 mRNA in the tumour cells by ISH, but also correlated the serum IL-6 levels with disease progression and survival of patients with ESCC. The serum levels of IL-6 were significantly higher in patients with positive lymph node metastasis and the advanced tumour stages. By demonstrating a similar IL-6 mRNA expression in ESCC cell lines, our work suggests that this phenomenon could be a general one. In the present study, the incidence of IL-6Rα in pathological sections was 88.7%, and IL-6 mRNA was 37.5%. Although the incidence of IL-6Rα is much higher than that of IL-6 in pathological sections, expression of IL-6 may be induced or enhanced by external stimulators, such as n-BT or other carcinogens. Currently, we have shown that n-BT can induce or enhance IL-6 mRNA expression in cancer cell lines as assayed by ISH and semi-quantitative RT-PCR. Furthermore, the increase of IL-6 mRNA expression is associated with a rise of IL-6 content in the supernatant as assayed by ELISA. Though the clinical roles of n-BT are evaluated in an ongoing study, as mentioned above, our results indicate that IL-6 expression in cancer cells could be potentially up-regulated by some external stimulators, such as n-BT. Not only could IL-6 be a prognostic predictor for the aggressiveness of ESCC, but also could elevated serum IL-6 reflect the pathogenic significance in progression of the malignancy.

Our previous experience in the management of patients with advanced T4 ESCC has shown that removal of tumour-bearing mass followed by an aggressive concurrent radiochemotherapy could provide significant improvement to patients than surgery alone (Wang et al, 1996). Most patients who have a decrease of IL-6 level following successful oesophagectomy remained disease-free for 12–23 months. In patients with persistent high levels of serum IL-6 or a marked increase of serum IL-6 level within a short interval following surgery, recurrence and distant metastasis of cancer were frequently found. However, a mixed population of IL-6 expression cells (macrophage, plasma cells, lymphocytes and tumour cells) was only found in three cases (3.8%) of pathological sections. These data considered together with our ESCC cell line studies clearly demonstrate that ESCC cells can produce IL-6, and IL-6 expression in ESCC cells can be up-regulated by n-BT and, maybe, a yet to be determined physiological function.

The increased gene expression of IL-6 has been shown in different types of human cells (fibroblasts, monocytes, epithelial cells and endothelial cells) as a result of a number of different stimuli, including tumour necrosis factor (TNF), IL-1, epidermoid growth factor (EGF) viruses, endotoxin, diacylglycerol and phorbol ester (TPA) (Ray et al, 1988). An increased level of intracellular cAMP was suggested responsible for the induced synthesis of IL-6 in human fibroblasts and HeLa cells (Ray et al, 1988; Zhang et al, 1988). However, upon examination of TPA effect on ESCC cell lines, no induction of IL-6 expression was observed (data not shown). Instead, synthesis and release of IL-6 in ESCC cell lines can be stimulated by n-BT. This phenomenon has not been demonstrated previously and is somewhat surprising. In fact, effect of n-BT on signal transduction pathway of calmodulin and calcineurin together with its relationship to the gene activation has been well documented (Chow et al, 1997). As noted above, both elevated urinary butyrate concentration and IL-6 level were shown in the AIDS patients with immune impairment and significant weight loss (Stein et al, 1997). In a cancer patient who developed cachexia, the immune compromised condition is similar to that of AIDS patient at the terminal stage. Detection of the increased expression of IL-6 could be therefore anticipated in many cancer patients as their conditions deteriorated. There is a good reason then to believe that IL-6–IL-6R loop may play an important role in autocrine growth of tumour cells (Kawano et al, 1988; Miki et al, 1989; Blay et al, 1992; Ni and O'Neill, 1992; Kabir and Daar, 1995; Oka et al, 1996; Wojciechowska-Lacka et al, 1996; Seymour et al, 1997). Nonetheless, these have not been consistently found for all the cancer cells in a given tumour type (Baba et al, 1995; Yanagawa et al, 1995).

The impact of IL-6 on tumour cell proliferation remains to be clarified if this is the basis of tumour growth. It should be noted, however, that other explanations are possible. In fact, tumour progression is a concerted process including evasion of host immunosurveillance (Chow and Chen, 1995), tumour cell self-proliferation (Kawano et al, 1988; Miki et al, 1989; VanMeir et al, 1990; Watson et al, 1990; Ni and O'Neill, 1992; Eustace et al, 1993; Oka et al, 1996; Takeuchi et al, 1996), killing of the immune cells (Walker et al, 1997) and invasion of neighbouring tissues as well as the distant organs. Association of IL-6 with cell attachment, migration and invasion was recently demonstrated by Obata et al (1997) on human ovarian carcinoma. At the present time, our results showed that serum levels of IL-6 were frequently elevated in patients with ESCC (> 90%), and the high serum IL-6 levels (≥ 9 pg $ml^{-1}$) may indicate a poor prognosis. Expression of IL-6 in cancer cells could be up-regulated by n-BT, a short-chain fatty acid produced by intestinal bacteria, certainly could indicate more implication on the host immunosurveillance in intestine during disease progression. Although there is not yet a clear explanation for the clinical correlation between increased IL-6 expression in cancer cells and disease progression, the observation provides a focus for future studies to elucidate the mechanism by which expression of IL-6 was regulated pathologically.

### ACKNOWLEDGEMENTS

We would like to thank Jin-Ping Lin, Wen-Ser Tseng, and Li-Ling Yang, for their excellent technical assistance. This study was supported, in part, by grant from Committee on Chinese Medicine and Pharmacy, Department of Health, Executive Yuan, R. O. C. (CCMP87-RD-025) and Veterans General Hospital–Taipei (VGH87-331) to K.C.C., and, in part, by Lite-on Cultural Foundation (LF97MD01).

### REFERENCES

Baba M, Hasegawa H, Nakayabu M, Shimizu N, Suzuki S, Kamada N and Tani K (1995) Establishment and characteristics of a gastric cancer cell line (HuGC-OOHIRA) producing high levels of G-CSF, GM-CSF, and IL-6: the presence of autocrine growth control by G-CSF. *Am J Hematol* **49**: 207–215

Blay JY, Negrier S, Combaret V, Attali S, Goillot E, Merrouche Y, Mercatello A, Ravault A, Tourani JM and Moskovtchenko JF (1992) Serum level of interleukin 6 as a prognostic factor in metastatic renal cell carcinoma. *Cancer Res* **52**: 3317–3322

Castell JV, Gomez-Lechon MJ, David M, Horano T, Kishimoto T and Heinrich PC (1990) Acute phase responses of human hepatocytes: regulation of acute phase protein synthesis by IL-6. *Hepatology* **12**: 1179–1186

© 1999 Cancer Research Campaign

Chiu CF, Chow KC, Lin FM, Lin CK, Liu SM and Chen KY (1997) Expression of DNA topoisomerase IIα and multidrug resistance p-glycoprotein in acute leukemia. *Chin Med J (Taipei)* **60**: 184–190

Chow KC and Chen KY (1995) The role of Epstein–Barr virus infection and the immunocompetence of patients with nasopharyngeal cancers. *Therapeutic Radiol Oncol* **2**: 243–260

Chow KC, Nacilla JQ, Witzig TE and Li CY (1992) Is persistent polyclonal B lymphocytosis caused by Epstein–Barr virus? A study with polymerase chain reaction and in situ hybridization. *Am J Hematol* **41**: 270–275

Chow KC, Zhang JH, Chou SL, Tseng WS and Chen KY (1997) The application of virus in the diagnosis and therapy of tumor. *Therapeutic Radiol Oncol* **4**: 155–178

Eustace D, Han X, Gooding R, Rowbottom A, Riches P and Heyderman E (1993) Interleukin 6 (IL-6) functions as an autocrine growth factor in cervical carcinoma in vitro. *Gyneccol Oncol* **50**: 151–159

Gauldie J, Richards C, Harnish D, Lansdorp P and Baumann H (1987) Interferon β 2/BSF2 shares identity with monocyte derived hepatocyte stimulating factor and regulates the major acute phase protein in liver cells. *Proc Natl Acad Sci USA* **84**: 7251–7255

Inoue M, Minami M, Fujii Y, Matsuda H, Shirakura R and Kido T (1997) Granulocyte colony-stimulating factor and interleukin-6-producing lung cancer cell line, LCAM. *J Surg Oncol* **64**: 347–350

Kabir S and Daar GA (1995) Serum levels of interleukin-1, interleukin-6 and tumour necrosis factor-alpha in patients with gastric carcinoma. *Cancer Lett* **95**: 207–212

Kaplan EL and Meier P (1958) Nonparametric estimation from incomplete observations. *Am Stat Assoc J* **53**: 457–481

Katlic MR, Wilkins EW and Grillo HC (1990) Three decades of treatment of oesophageal squamous carcinoma at the Massachusetts General Hospital. *J Thorac Cardiovasc Surg* **99**: 929–938

Kawano M, Hirano T, Matsuda T, Taga T, Horii Y, Iwato K, Asaoku H, Tang B, Tanabe O and Tanaka H (1988) Autocrine generation and requirement of BSF-2/IL-6 for human multiple myeloma. *Nature* **332**: 83–85

Kishimoto T (1990) The biology of interleukin 6. *Blood* **74**: 1–10

Lee JD, Sievers TM, Skotzko M, Chandler CF, Morton DL, McBride WH and Economou JS (1992) Interleukin 6 production by human melanoma cell lines. *Lymphokine Cytokine Res* **11**: 161–166

Liu CC, Fahn HJ, Li WY, Wu YC, Huang MH and Wang LS (1998) Lymph node metastasis in squamous cell carcinoma of the intrathoracic esophagus. *Chin Med J (Taipei)* **61**: 77–84

Miki S, Iwano M, Miki Y, Yamamoto M, Tang B,Yokokawa K, Sonoda T, Hirano T and Kishimoto T (1989) Interleukin 6 (IL-6) functions as an in vitro autocrine growth factor in renal cell carcinomas. *FEBS Lett* **250**: 607–610

Ni K and O'Neill HC (1992) Proliferation of the BCL1 B cell lymphoma induced by IL-4 and IL-5 is dependent on IL-6 and GM-CSF. *Immunol Cell Biol* **70**: 315–321

Obata NH, Tamakoshi K, Shibata K, Kikkawa F and Tomoda Y (1997) Effects of interleukin-6 on in vitro cell attachment, migration and invasion of human ovarian carcinoma. *Anticancer Res* **17**: 337–342

Oka M, Yamamoto K, Takahashi M, Hakozaki M, Abe T, Iizuka N, Hazama S, Hirazawa K, Hayashi H, Tangoku A, Hirose K, Ishihara T and Suzuki T (1996) Relationship between serum levels of interleukin 6, various disease parameters, and malnutrition in patients with oesophageal carcinoma. *Cancer Res* **56**: 2776–2780

Peto R and Pike MC (1973) Conservatism of the approximation $\Sigma$ (O–E)$^2$/E in the log-rank test for survival data or incidence data. *Biometrics* **29**: 579–584

Ray A, Tatter SB, May LT and Sehgal PB (1988) Activation of the human '$\beta_2$-interferon/hepatocyte-stimulating factor/interleukin 6' promoter by cytokines, viruses, and second messenger agonists. *Proc Natl Acad Sci USA* **85**: 6701–6705

Reichner JS, Mulligan JA, Palla ME, Hixson DC, Albina JE and Bland K (1996) Interleukin-6 production by rat hepatocellular carcinoma cells is associated with metastatic potential but not with tumorigenicity. *Arch Surg* **131**: 360–365

Scambia G, Testa U, Benedetti Panici P, Foti E, Martucci R, Gadducci A, Perillo A, Facchini V, Peschle C and Mancuso S (1995) Prognostic significance of interleukin 6 serum levels in patients with ovarian cancer. *Br J Cancer* **71**: 354–356

Seymour JF, Talpaz M, Hagemeister FB, Cabanillas F and Kurzrock R (1997) Clinical correlates of elevated serum levels of interleukin 6 in patients with untreated Hodgkin's disease. *Am J Med* **102**: 21–28

Stein TP, Koerner B, Schluter MD, Leskiw MJ, Gaprindachvilli T, Richards EW, Cope FO and Condolucci D (1997) Weight loss, the gut and the inflammatory response in Aids patients. *Cytokine* **9**: 143–147

Strassmann G, Fong M, Kenney JS and Jacob CO (1992*a*) Evidence for the involvement of interleukin 6 in experimental cancer cachexia. *J Clin Invest* **89**: 1681–1684

Strassmann G, Jacob CO, Evans R, Beall D and Fong M (1992*b*) Mechanism of experimental cancer cachexia. Interaction between mononuclear phagocytes and colon-26 carcinoma and its relevance to IL-6-mediated cancer cachexia. *J Immunol* **148**: 3674–3678

Stephanou A, Knight RA, Annicchiarico-Petruzzelli M, Finazzi-Agro A, Lightmann SL and Melino G (1992) Interleukin-1 beta and interleukin-6 mRNA are expressed in human glioblastoma and neuroblastoma cells respectively. *Funct Neurol* **7**: 129–133

Tabibzadeh SS, Poubouridis D, May LT and Sehgal PB (1989) Interleukin 6 immunoactivity in human tumors. *Am J Pathol* **135**: 427–433

Taga T, Hibi M, Hirata Y, Yamasaki K, Yasukawa K, Matsuda T, Hirano T and Kishimoto T (1989) Interleukin-6 triggers the association of its receptor with a possible signal transducer, gp130. *Cell* **58**: 573–581

Takeuchi E, Ito M, Mori M, Yamaguchi T, Nakagawa M, Yokota S, Nishikawa H, Sakuma-Mochizuki J, Hayashi S and Ogura T (1996) Lung cancer producing interleukin-6. *Intern Med* **35**: 212–214

Takizawa H, Ohtoshi T, Ohta K, Yamashita N, Hirohata S, Hirai K, Hiramatsu K and Ito K (1993) Growth inhibition of human lung cancer cell lines by interleukin 6 in vitro: a possible role in tumor growth via an autocrine mechanism. *Cancer Res* **53**: 4175–4181

Tamura S, Fujimoto-Ouchi K, Mori K, Endo M, Matsumoto T, Eden H, Tanaka Y, Ishitsuka H, Tokita H and Yamaguchi K (1995) Involvement of human interleukin 6 in experimental cachexia induced by a human uterine carcinoma xenograft. *Clin Cancer Res* **1**: 1353–1358

Tanner J and Tosato G (1991) Impairment of natural killer functions by interleukin 6 increases lymphoblastoid cell tumorigenicity in athymic mice. *J Clin Invest* **88**: 239–247.

VanMeir E, Sawamura Y, Diserens AC, Hamou MF and de Tribolet N (1990) Human glioblastoma cells release interleukin 6 in vitro and in vivo. *Cancer Res* **50**: 6683–6688

Walker PR, Saas P and Dietrich PV (1997) Role of Fas ligand (CD95L) in immune ESCCape. *J Immunol* **158**: 4521–4524

Wang GG, Witek-Giannotti J, Hewick RM, Clark SC and Ogawa M (1988) Interleukin 6: identification as a hematopoietic colony-stimulating factor. *Behring Inst Mitt* **83**: 40–47

Wang LS, Huang MH, Huang BS and Chien KY (1992) Gastric substitution for resectable carcinoma of the esophagus. An analysis of 368 cases. *Ann Thorac Surg* **53**: 289–294

Wang LS, Chi KH, Hu MH, Fahn HJ and Huang MH (1996) Management for patients with advanced T4 epidermoid carcinoma of the esophagus. *J Surg Oncol* **62**: 22–29

Wang LS, Lin HY, Chang CJ, Fahn HJ, Huang MH and Lin CF (1998) Effects of en bloc esophagectomy on nutritional and immune status in patients with esophageal carcinoma. *J Surg Oncology* **67**: 90–98

Watson JM, Sensintaffar JL, Berek JS and Martinez-Maza O (1990) Constitutive production of interleukin 6 in viro: a possible role in tumor growth via an autocrine mechanism. *Cancer Res* **50**: 6959–6965

Wojciechowska-Lacka A, Matecka-Nowak M, Adamiak E, Lacki JK and Cerkaska-Gluszak B (1996) Serum levels of interleukin-10 and interleukin-6 in patients with lung cancer. *Neoplasma* **43**: 155–158

Wu CW, Wang SR, Chao MF, Wu TC, Lui WY, P'eng FK and Chi CW (1996) Serum interleukin-6 levels reflect disease status of gastric cancer. *Am J Gastroenterol* **91**: 1417–1422

Wu YK, Chen PT, Fang J and Lin SS (1980) Surgical treatment of oesophageal carcinoma. *Am J Surg* **139**: 805–809

Yamasaki K, Taga T, Hirata Y, Yawata H, Kawanishi Y, Seed B, Taniguchi T, Hirano T and Kishimoto T (1988) Cloning and expression of the human interleukin-6 (BFS-2/IFNB2) receptor. *Science* **241**: 825–828

Yamashita S, Ogawa M, Abe T, Yamashita J, Sakamoto K, Niwa H and Yamamura K (1994) Group II phospholipase A2 in invasive gastric cancer cell line is induced by interleukin 6. *Biochem Biophys Res Commun* **198**: 878–884

Yanagawa H, Sone S, Takahashi Y, Haku T, Yano S, Shinohara T and Ogura T (1995) Serum levels of interleukin 6 in patients with lung cancer. *Br J Cancer* **71**: 1095–1098

Zhang Y, Lin JX and Vilcek J (1988) Synthesis of interleukin 6 (interferon-$\beta_2$/B cell stimulatory factor 2) in human fibroblasts is triggered by an increase in intracellular cyclic AMP. *J Biol Chem* **263**: 6177–6182

*© 1999 Cancer Research Campaign*
